# The association between metabolic syndrome and the occurrence of nephrolithiasis in HIV-infected patients

**DOI:** 10.1186/1758-2652-13-S4-P88

**Published:** 2010-11-08

**Authors:** IM Dumitru, S Rugina, G Sotila, E Dumitru, CN Rugina

**Affiliations:** 1 Ovidius University, Constanta, Romania

## Introduction

Although HAART therapy has radically changed the prognosis of HIV infected patients, decreasing their morbidity and mortality, the ARV medication produced other clinical manifestations due to their toxicity. For many years there was no data in the literature attesting link between metabolic syndrome and renal disease development. Recent epidemiological studies have found that patients with metabolic syndrome have a high risk of developing chronic kidney disease: chronic renal failure and nephrolithiasis.

## Purpose of the study

Evaluation of the relationship between metabolic syndrome and the occurrence of nephrolithiasis in HIV infected patients.

## Methods

The study involved 112 patients with known HIV-AIDS infection and metabolic syndrome in the evidence of the Regional HIV Centre Constanta, Romania which were compared with 100 matched control group. The parameters analyzed were: demographic characteristics, weight, height, body mass index, blood pressure, medical history, examination of urine, urea, creatinine, Na, K, Cl, Mg, P, total serum calcium, glucose, serum triglycerides, LDH cholesterol, HDL cholesterol, CD4+ lymphocytes, HIV-RNA. Nephrolithiasis was diagnosed by ultrasound examination. No patient had a history of treatment with Indinavir.

## Results

Of the 112 patients studied, 67 patients developed nephrolithiasis (59.82%) in comparison with only 24% in the control group (p < 0.01). The age of these patients was between 20 and 67 years, with a mean age of 43.5, sex ratio F:M = 1.09. The predominant form was bilateral (69%) and asymptomatic (58%). The analysis of kidney stones revealed that the major component was uric acid (48%). Ultrasound revealed hydronephrosis in 18 patients (26.8%). Urinary tract infection was diagnosed in 23 patients (34.3%); the most common etiology was E. coli (39%). PCR HIV-RNA was undetectable in 90 patients (80.3%), and 46 patients had a CD4+ cell count > 500 cells/mm^3^ and only 10 patients CD4+ < 200 cells/mm^3^. The majority of patients (55.3%) received 2INRT and PI/r as ARV treatment.

## Conclusions

In HIV infected patients with metabolic syndrome under ARV treatment, nephrolithiasis is 2.5 times more frequent compared to general population. The presence of kidney stones is a risk factor for developing hydronephrosis and urinary tract infection. Because patients with HIV infection and chronic renal failure have a decreased survival, the screening for nephrolithiasis is mandatory in these patients.

